# State-dependent effects of responsive neurostimulation depend on seizure localization

**DOI:** 10.1093/brain/awae240

**Published:** 2024-07-25

**Authors:** Sharon Chiang, Ankit N Khambhati, Thomas K Tcheng, Audra Plenys Loftman, Nicholas R Hasulak, Emily A Mirro, Martha J Morrell, Vikram R Rao

**Affiliations:** Department of Neurology and Weill Institute for Neurosciences, University of California, San Francisco, San Francisco, CA 94158, USA; Department of Physiology and the Kavli Institute for Fundamental Neuroscience, University of California, San Francisco, CA 94158, USA; Department of Neurological Surgery, University of California, San Francisco, San Francisco, CA 94143, USA; NeuroPace Inc, Mountain View, CA 94043, USA; Clinical Research Consultant, Menlo Park, CA 94025, USA; Phoenix Research Consulting, LLC, Gilbert, AZ 85233, USA; SynchNeuro, Inc, Philadelphia, PA 19106, USA; NeuroPace Inc, Mountain View, CA 94043, USA; Department of Neurology and Weill Institute for Neurosciences, University of California, San Francisco, San Francisco, CA 94158, USA

**Keywords:** brain-responsive neurostimulation, state-dependence, closed-loop stimulation, epilepsy, mesiotemporal epilepsies, state-space modelling

## Abstract

Brain-responsive neurostimulation (RNS) is firmly ensconced among treatment options for drug-resistant focal epilepsy, but over a quarter of patients treated with the RNS^®^ System do not experience meaningful seizure reduction. Initial titration of RNS therapy is typically similar for all patients, raising the possibility that treatment response might be enhanced by consideration of patient-specific variables. Indeed, small, single-centre studies have yielded preliminary evidence that RNS System effectiveness depends on the brain state during which stimulation is applied. The generalizability of these findings remains unclear, however, and it is unknown whether state-dependent effects of responsive neurostimulation are also stratified by location of the seizure onset zone where stimulation is delivered. We aimed to determine whether state-dependent effects of the RNS System are evident in the large, diverse, multi-centre cohort of RNS System clinical trial participants and to test whether these effects differ between mesiotemporal and neocortical epilepsies.

Eighty-one of 256 patients treated with the RNS System across 31 centres during clinical trials met the criteria for inclusion in this retrospective study. Risk states were defined in relation to phases of daily and multi-day cycles of interictal epileptiform activity that are thought to determine seizure likelihood. We found that the probabilities of risk state transitions depended on the stimulation parameter being changed, the starting seizure risk state and the stimulated brain region. Changes in two commonly adjusted stimulation parameters, charge density and stimulation frequency, produced opposite effects on risk state transitions depending on seizure localization. Greater variance in acute risk state transitions was explained by state-dependent responsive neurostimulation for bipolar stimulation in neocortical epilepsies and for monopolar stimulation in mesiotemporal epilepsies.

Variability in the effectiveness of RNS System therapy across individuals may relate, at least partly, to the fact that current treatment paradigms do not account fully for fluctuations in brain states or locations of simulation sites. State-dependence of electrical brain stimulation may inform the development of next-generation closed-loop devices that can detect changes in brain state and deliver adaptive, localization-specific patterns of stimulation to maximize therapeutic effects.

## Teaser

Chiang *et al.* study the effects of closed-loop brain-responsive neurostimulation via the RNS System on drug-resistant focal epilepsy. They show that stimulation parameters effective at reducing seizures in one brain state can be counterproductive in another and that the location of the stimulation site matters.

## Introduction

Brain-responsive neurostimulation with the RNS^®^ System is an established therapy for certain forms of drug-resistant focal epilepsy,^1^ but its effectiveness across patients is highly variable.^2,3^ In clinical trials of the RNS System, a third of patients achieved more than 90% reduction in seizure frequency, but a similar fraction of patients were non-responders, experiencing less than 50% reduction in seizure frequency.^2^ This outcome variability is not explained by obvious clinical characteristics^4,5^ but may relate to intrinsic features of brain networks,^6-8^ including the connectivity of nodes where stimulation is delivered.^9,10^ Effectiveness of RNS therapy may also depend on the momentary state of brain networks; for example, the same stimulation parameters that reduce seizures during a high seizure risk state could be counterproductive during a low seizure risk state by promoting transition to a high-risk state.^11^ Observed outcomes with the RNS System may reflect the net of opposing effects during seizure risk state cycling^12-14^ and may underestimate the potential of this therapy.

By virtue of its closed-loop design, the RNS System is intrinsically dynamic, delivering stimulation only in response to detections of epileptiform brain activity. Yet, parameters programmed on the device are static during weeks- to months-long intervals between outpatient clinic visits, while seizure network states may continue to evolve over similar timescales.^15^ In the contemporary paradigm for device programming during patient clinic visits, stimulation is not enabled until the device is tuned to detect seizures reliably. Stimulation is then enabled, usually with initial parameters (e.g. charge density 0.5 μC/cm^2^) based on experience from the RNS System clinical trials. In subsequent visits, the charge density is increased incrementally up to 3 μC/cm^2^. If seizures persist, higher charge densities can be trialled, or changes can be made to other parameters, such as stimulation frequency, burst duration, or, less often, pulse width. Beyond recommended initial settings, however, few guidelines exist to help clinicians titrate stimulation parameters, and device programming remains largely empiric. Whether stimulation parameters should be tailored based on location of the seizure onset zone (e.g. mesiotemporal versus neocortical) is also unknown.

In a preliminary study of a small group of patients with mixed mesiotemporal and neocortical epilepsies, we previously found that the effects of responsive neurostimulation depended on whether patients were in a high- or low-risk state.^11^ Another study found that the effectiveness of RNS therapy is greater when stimulation is preferentially delivered during low-risk states.^16^ Both of these single institutional studies have been limited in their ability to consider separately the mesiotemporal and neocortical groups due to small sample sizes. Furthermore, RNS System programming was performed primarily by a single or small number of operators, limiting generalizability and introducing potential biases. Ideally, the hypothesis that there are state-dependent effects of RNS therapy would be best tested in a larger, multi-centre cohort in which diverse epilepsies and diverse programming approaches are represented. Generalizable principles uncovered through such an analysis could inform prospective studies and the development of next-generation neurostimulation systems.

We therefore conducted a retrospective analysis of a large, diverse cohort of patients treated with a wide range of stimulation parameters at multiple institutions during the RNS System clinical trials. We aimed to quantify the impact of responsive neurostimulation on seizure risk transitions beyond the natural rate of transitions that occurs in the absence of stimulation.^14,17-19^ We hypothesized that RNS System stimulation parameters differentially influence the probability of transitioning between high- and low-risk states, and that mesiotemporal and neocortical epilepsies exhibit distinct patterns of stimulation-dependent state transition probabilities.

## Materials and methods

### Study population

We conducted a retrospective analysis of intracranial EEG data from a subset of the 256 adults with drug-resistant focal epilepsy involving one to two seizure foci who participated in the RNS^®^ System clinical trials (NeuroPace Inc.) between 19 January 2004 and 11 November 2008 (NCT00079781, NCT00264810, NCT00572195) (Table 1). All patients provided written informed consent. All study protocols were approved by the US Food and Drug Administration (FDA) and institutional review boards of participating investigation sites. The RNS System stores continuous hourly counts of interictal epileptiform activity (IEA) and long episodes (LE), detections of epileptiform activity that persist beyond a pre-specified duration and that are often a reliable proxy for electrographic seizures. To control for the potential influence of changes in detection settings on LE counts, only programming epochs for which detection settings and LE duration were held constant were included.^13,20,21^ Electrocorticograms stored by the RNS System due to occurrence of LEs were visually reviewed to determine how reliably LEs corresponded to electrographic seizures.^22^ Only epochs for which >90% of LEs detected by the RNS System corresponded to electrographic seizures^13^ and with a minimum of 30 days in the epoch were included. Patients were included if they had at least one programming epoch meeting the criteria and had (i) both leads placed in mesiotemporal locations; (ii) both leads placed in neocortical locations; or (iii) one lead placed in a mesiotemporal location and the other lead placed in a neocortical location but with one lead inactivated during both bursts of the first delivered stimulation therapy. Patients with mixed neocortical and mesiotemporal stimulation during therapy 1 were excluded. The RNS System can deliver up to five successive ‘therapies’ (each comprising two consecutive bursts of electrical pulses) during a detection episode. Because stimulation parameters from therapy 1 are those that are most often varied in practice and the majority of episodes result in just one responsive therapy, such that nearly 90% of stimulations delivered by the RNS System are during therapy 1 and about 10% of stimulations are delivered as therapies 2–5 (NeuroPace, Inc., unpublished data), we focused on stimulation parameters from therapy 1 in this analysis.

**Table 1:** Clinical characteristics

Clinical characteristic	Median/Proportion	Mesial temporal stimulation (*n* = 49)	Neocortical stimulation (*n* = 32)	*P*-value
Age at time of implant, years	35.0 (24.0–43.0)	37.0 (30.0–45.0)	28.0 (22.8–37.8)	0.02^a^
Sex				
Female	41 (50.6%)	24 (49.0%)	17 (53.1%)	0.9^b^
Male	40 (59.4%)	25 (51.0%)	15 (46.9%)	
Seizure frequency, daily				
Electrographic seizures	1.0 (0.3–7.0)	0.9 (0.3–6.9)	1.4 (0.5–7.3)	0.27^a^
Focal impaired aware and generalized tonic clonic seizures	0.1 (0.04–0.4)	0.1 (0.05–0.3)	0.5 (0.3–1.6)	<0.001^a^
Focal aware seizures	0.0 (0.0–0.1)	0.0 (0.0–0.1)	0.09 (0.0–0.5)	<0.001^a^

For proportions, number (%) are shown. For continuous variables, median (Q1–Q3) are shown.

^a^Wilcoxon rank sum test.

^b^Two-tailed test of proportions.

### Data preprocessing

Hourly LE counts and the average charge density, stimulation frequency, burst duration, and pulse width across the two bursts within therapy 1 (T1B1 and T1B2) for each corresponding hour were analysed (Supplementary Fig. 1). For cases where the second burst in the therapy was disabled or charge density was zero, the charge density, stimulation frequency, burst duration, and pulse width during the first therapy burst were used. Because stimulation parameter changes can be programmed at any time of day, if a stimulation parameter change occurred partway through an hourly time bin, the stimulation parameter at the beginning of the hourly time bin was imputed through the end of the hour. For hours during which no detections were made, no episodes with stimulation occurred, or stimulation was disabled, the charge density, frequency, burst duration, and pulse width were set to zero. Time series were concatenated across continuous segments within each patient, and risk state transitions for each patient were modelled separately.

### Statistical analysis

Statistical analysis was conducted in R version 4.1.2. Risk states were inferred by considering both multidien/circadian cycles in IEA, which are a measure of interictal activity, as well as LEs, which are a measure of electrographic seizures. IEA counts were normalized via a z-transformation and bandpass filtered to the following ranges, using a first-order Butterworth filter: Circadian (0.8–1.2 of 24 h, or 19.2–28.8 h), 7 days (0.8–1.2 of 7 days, or 5.6–8.4 days), 15 days (0.8–1.2 of 15 days, or 12–18 days), 20 days (0.8–1.2 of 20 days, or 16–24 days) and 30 days (0.8–1.2 of 30 days, or 24–36 days), as these multidien periodicities have been found to be prevalent among patients with focal epilepsy.^23^ The instantaneous phase of IEA counts within each bandpass frequency range was calculated via the Hilbert transform. Because seizures have been found to be generally more prevalent during the rising phase of multidien IEA cycles,^13^ and because values of phase between -π and 0 computed from the Hilbert transform correspond with the rising phase IEA and values between 0 and π correspond with the falling phase of IEA, phase was shifted by π, such that higher values of phase correspond to the peak of the upgoing phase of IEA for each frequency band. Instantaneous shifted phases of circadian and multidien frequency bands and log-transformed LE counts for each epoch were subsequently transformed into orthogonal components via principal components analysis (PCA). To consider the possibility that more than two risk states may exist, two- and three-state multivariate Gaussian hidden Markov models (HMMs) were then fit via the depmixS4 library to the first five principal components (PCs) to infer latent seizure risk states.^24^ The number of PCs was chosen based on the minimum number of PCs to capture at least 80% of variance in the IEA phase and LE count in all patients. Identifiability, which refers to the ability to uniquely identify parameters in a model (e.g. due to label-switching in HMMs, wherein the label assigned to each state may ‘switch’ between runs), was enforced in the three-state HMM by setting the third state to the state with the largest mean number of LE over all epochs, the second state to that with the greatest positive mean value of the shifted phase over all circadian and multidien cycles, and the first state to the remaining state. In the two-state HMM, identifiability was enforced by setting the second state to the state with the largest mean number of LEs over all epochs for the patient and the first state to the remaining state. The number of states was subsequently selected based on minimization of the Akaike information criterion (AIC) as a measure of model fit. Correspondence of identified seizure risk states to times of higher risk for seizures and epileptiform activity was checked through Wilcoxon rank-sum tests comparing the number of LEs per hour and number of IEA per hour between identified risk states at the 0.05 level of significance.

To capture the impact of neurostimulation parameters on state transition rates beyond the baseline rate of transition in the absence of stimulation therapy, conditional on each latent state, a generalized linear mixed effects (GLME) model was fit to the probability of transitioning or remaining in a high-risk state in the subsequent hour. Average charge density, stimulation frequency, pulse width, burst duration across the two therapy 1 bursts, and number of stimulations per hour were treated as fixed effects and categorized as shown in Table 2, with patient and number of days since RNS System implantation treated as random effects. Bipolar neocortical stimulation was used in seven patients; due to low sample size to estimate the associations with parameter categories, stimulation parameters were treated as continuous variables. Continuous variables were centred and scaled before fitting. For GLME coefficients, 95% confidence intervals are reported for state-dependent associations between stimulation parameters and the probability of remaining in/transitioning to a low-risk state for neocortical and mesiotemporal stimulation groups. Monopolar and lead-to-lead stimulation were evaluated as a single group and bipolar stimulation evaluated as a separate group due to presumed differences in the volume of tissue activated from broad (monopolar) compared to localized (bipolar) stimulation fields.^25,26^ Only configurations where both therapy 1 bursts were either both monopolar/lead-to-lead or both bipolar were considered. Mixed stimulation pathways (i.e. with different classes of stimulation in different therapy bursts) were excluded. Grouped bipolar stimulation in both therapy bursts, which involves groupings of two anode contacts and two cathode contacts per lead, had data satisfying the inclusion/exclusion criteria in only six patients and was not considered in this analysis due to the low sample size. Hybrid monopolar/bipolar stimulation was excluded from the analysis. For bipolar neocortical stimulation, only fixed effects were included due to the low sample size for estimating random effects. Significant fixed effects were identified at the 0.05 level, with family-wise error rate control through the Holm correction for multiple comparisons.^27^

**Table 2:** Ranges of average therapy 1 stimulation parameters evaluated

	Charge density	Stimulation frequency	Pulse width	Burst duration
Baseline (manufacturer-recommended initial settings)	≤0.5 μC/cm^2^	200 Hz	160 μs	100 ms
Evaluated ranges	(0.5, 3] μC/cm^2^	≤20 Hz	<160 μs	<100 ms
	(3, 4] μC/cm^2^	(20, 100) Hz	>160 μs	(100, 500] ms
	(4, 5] μC/cm^2^	100 Hz	–	>500 ms
	(5, 6] μC/cm^2^	(100, 200) Hz	–	–
	(6, 7] μC/cm^2^	>200 Hz	–	–
	>7 μC/cm^2^	–	–	–

## Results

### Study sample

From a total of 256 adults with drug-resistant focal epilepsy who participated in the RNS System clinical trials^28^ across 31 centres, 81 (31.6%) met the inclusion/exclusion criteria. These patients collectively had a total of 300 stable programming epochs satisfying the inclusion criteria (median 3 epochs/patient; Q1–Q3, 1–5 epochs/patient) and a total of 2 212 024 person-hours of treatment with the RNS System.

Demographic characteristics are listed in Table 1. The median age of implantation with the RNS System was 35 years, ranging from 18–66 years. There were 49 (60.5%) patients with mesiotemporal stimulation and 32 (39.5%) with neocortical stimulation. Patients had a median daily electrographic seizure frequency of 1.0 LEs/day (Q1–Q3 0.3–7.0, range 0.0028–499.7). Patients with neocortical stimulation were significantly younger with higher self-reported clinical seizure frequencies, although electrographic seizure frequencies did not significantly differ between patients with neocortical and mesiotemporal stimulation (Table 1). Among patients with mesiotemporal stimulation, 37 (75.5%) had bilateral foci, seven (14.3%) had a left temporal focus, three (6.1%) had a right temporal focus and two (4.0%) had a left temporal and a left hemispheric neocortical focus. For both patients with both temporal and neocortical foci, the neocortical focus was not stimulated in bursts 1 and 2 of the first therapy. The median daily clinical seizure frequency was 0.2 seizures/day (Q1–Q3 0.06–0.6, range 0–13.9) for focal impaired aware and bilateral tonic-clonic seizures and 0.003 seizures/day (Q1–Q3 0.0–0.2, range 0–11.4) for focal aware seizures. Of the 2 212 024 person-hours analysed, 1 947 904 h (88.1%) had stimulation delivered, with a median of 92.1% of hours with stimulation per patient (Q1–Q3, 77.7–97.3%, range 11.4–99.9%). There was a median of 4739 h analysed per stable recording epoch (Q1–Q3 2269–10 070 h, range 720–43 032 h) and a median of 18 446 h analysed per patient over all epochs (Q1–Q3 8664–48 923 h, range 744–78 656 h). Stimulation configurations analysed are shown in Supplementary Table 1. The distributions of stimulation parameters in the first therapy employed in each stimulation configuration are shown in Supplementary Fig. 2. Table 2 shows the ranges of stimulation parameters that were compared relative to the recommended RNS System initial stimulation settings (charge density, 0.5 μC/cm^2^; pulse width, 160 μs; stimulation frequency, 200 Hz; burst duration, 100 ms).^1^ Univariate associations between parameter values and state transitions are shown in Supplementary Tables 3–5 for models treating stimulation parameters as categorical variables.

### Latent state inference

**Figure 1:**
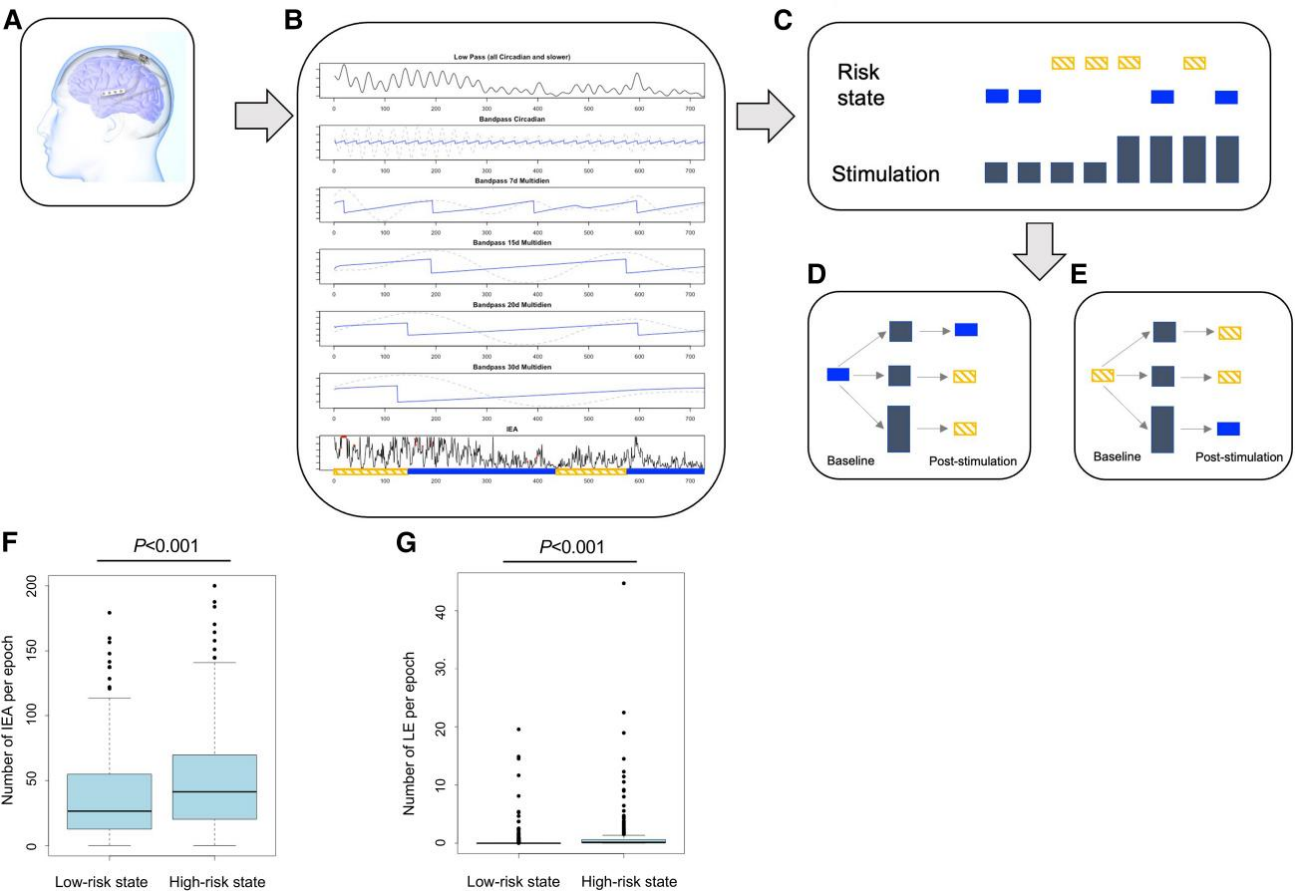
**Representative patient demonstrating the process for extracting latent seizure risk states from interictal epileptiform activity (IEA) and long episodes (LEs) and estimating the association of stimulation parameters with hourly risk state transitions**. In this example, the patient undergoes three state transitions over the course of the month shown, with seizures occurring during the first high-risk state (18 LEs in 143 h) and towards the beginning and end of the second low-risk state (nine LEs in 292 h). (**A**) Patients underwent implantation with brain-responsive neurostimulation (RNS) using the RNS System for the treatment of epilepsy as part of the RNS System clinical trials. (**B**) Raw IEA and LE counts were extracted (*bottom row*) and IEA counts were filtered (normalized) into circadian and multidien periodicities (*top five rows*; 7 day, 15 day, 20 day and 30 day). Here, the solid lines in *rows 2–6* indicate shifted phase and the dotted lines indicate filtered IEA counts. A principal component analysis (PCA) was performed and a Gaussian Hidden Markov Model fit to the first five principal components to identify seizure risk states. In the *bottom row*, the extracted seizure risk states are superimposed on raw IEA counts (black line) and long episodes (red filled circles); yellow dashed rectangles denote times in a high-risk state; blue solid rectangles denote times in a low-risk state. (**C**) A generalized linear mixed effects model was then fit to estimate the association between stimulation parameter values (depicted as grey bars of different heights) and the probability of transitioning to or remaining in a low-risk state in the subsequent hour, contingent on whether each patient was currently in a low-risk (**D**; blue solid rectangle) or high-risk (**E**; yellow dashed rectangle) state. As shown in **F** and **G**, inferred seizure risk states had a higher median number of IEA per epoch (**F**) as well as LEs per epoch (**G**).

A two-state HMM minimized the AIC, providing a better model fit than the three-state HMM. Figure 1 shows the inference on latent seizure risk states for an epoch for a sample patient, which demonstrates model identification of high-risk states as not simply those with LEs and low-risk states as those without LEs, but rather identification of high-risk states as those with greater likelihood of an LE per unit time (18 LEs in 143 h during the first low-risk state and nine LEs in 292 h during the second high-risk state). Overall, high-risk states had a significantly greater mean number of LEs per hour (0.3 ± 1.7 versus 0.8 ± 2.4, *P* < 0.001) and mean number of IEA per hour (40.9 ± 34.6 versus 54.5 ± 41.3, *P* < 0.001) than low-risk states (Fig. 1F and G). As shown in Fig. 1, high-risk states (yellow) were identified either as times when rising phases of IEA circadian and multidien cycles overlapped with LEs if LEs occurred more during the rising phase or times when the falling phase of IEA cycles overlapped with LEs if LEs occurred more during the falling phase of IEA.

### Monopolar mesiotemporal stimulation

**Figure 2:**
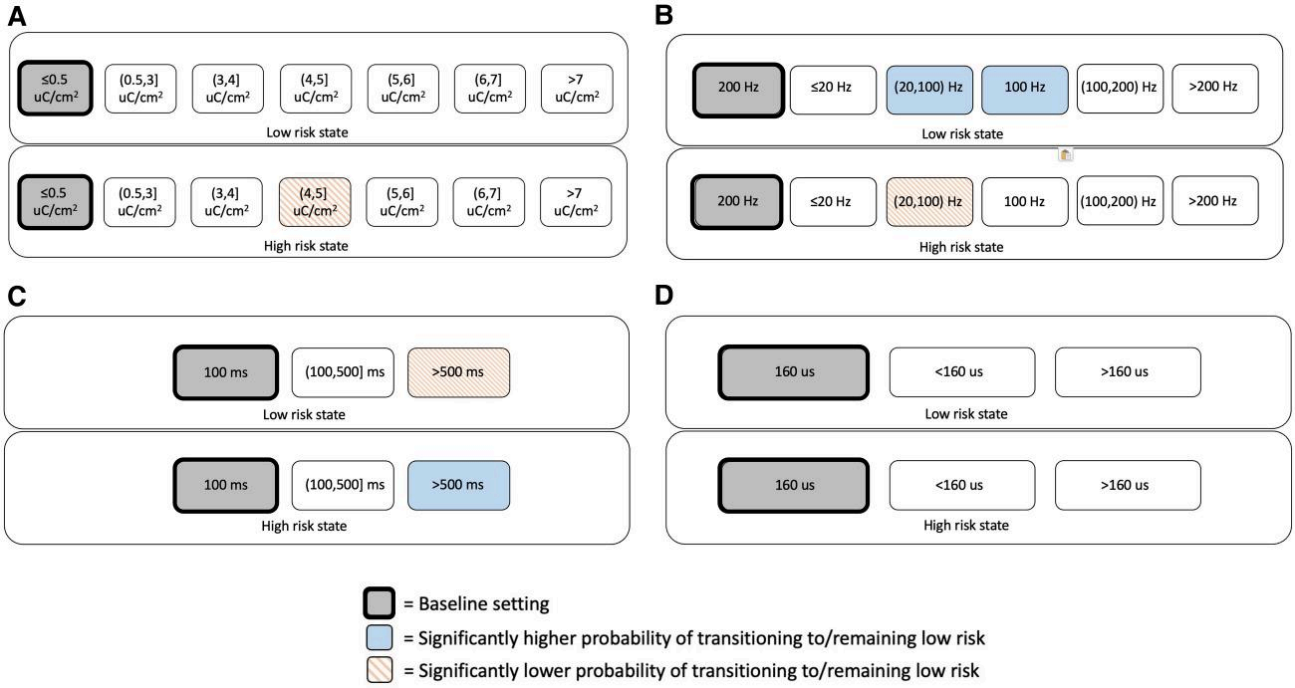
**For monopolar mesial temporal stimulation, associations of brain-responsive neurostimulation (RNS) using RNS System stimulation parameter settings with the probability of transitioning to or remaining in a low-risk state, conditional on current state.** (**A**) Charge density, (**B**) stimulation frequency, (**C**) burst duration and (**D**) pulse width. Baseline setting is shown in grey (bolded rectangles). Blue solid shading indicates settings found to have a significantly higher probability of transitioning to or remaining in a low-risk state relative to the baseline setting. Orange dashed shading indicates settings found to have a significantly lower probability of transitioning to or remaining in a low-risk state relative to the baseline setting. No significant associations were present for pulse width. Parameter ranges over the two bursts in the first therapy: charge densities of 0.40–7.15 μC/cm^2^, stimulation frequencies of 4–333.3 Hz, burst durations of 100–2000 ms and pulse widths of 80–240 μs.

Random effects for GLME models are presented in Supplementary Table 2 and show the variability in hourly state transitions attributed to differences between patients and time since RNS System implantation. Twenty patients with mesiotemporal leads underwent monopolar or lead-to-lead stimulation during RNS therapy. Table 3 shows the association of RNS System stimulation parameters with the probability of remaining/transitioning to a low-risk state in monopolar mesiotemporal stimulation, relative to the baseline state transition rate. The various charge densities examined did not demonstrate a significant impact on the probability of remaining in/transitioning to a low-risk state in the subsequent hour beyond the baseline state transition rate. Use of >20 to <100 Hz stimulation during low-risk states was associated with an improved probability of remaining low-risk compared with 200 Hz stimulation (*P* = 0.001). In contrast, in high-risk states, use of >20 to <100 and 100 Hz stimulation was associated with a lower probability of transitioning to a low-risk state (*P* < 0.001 and *P* < 0.001, respectively; Fig. 2B). Long burst durations above 500 ms (in this sample, a burst duration of 2000 ms) were effective in high-risk states (*P* < 0.001) but counterproductive when patients were in low-risk states (*P* < 0.001; Fig. 2C). There was no significant difference between 160 μs, <160 μs and >160 μs pulse widths in association with subsequent transitions to low-risk states (Fig. 2D). Overall, responsive neurostimulation using monopolar mesiotemporal stimulation explained 5% of the variance in hourly state transitions during low-risk states and 13% during high-risk states, corresponding to small and medium effect sizes, respectively.^29^

**Table 3:** Mesial temporal versus neocortical state-dependent parameter associations: monopolar or lead-to-lead stimulation

Mesial temporal leads (*n* = 20)				
	Low-risk state (R^2^_marginal_ = 0.05, R^2^_conditional_ = 0.37, AIC 39 218.3)		High-risk state (R^2^_marginal_ = 0.13, R^2^_conditional_ = 0.43, AIC 37 961.9)	
	Coefficient estimate (SE)	Adjusted *P*-value	Coefficient estimate (SE)	Adjusted *P*-value
Intercept	18.09 (9.23–35.44)	<0.001	0.06 (0.03–0.12)	<0.001
Charge density (0.5, 3] μC/cm^2a^	1.00 (0.68–1.48)	0.99	0.90 (0.66–1.24)	0.99
Charge density (3, 4] μC/cm^2a^	1.21 (0.80–1.82)	0.99	0.77 (0.55–1.07)	0.99
Charge density (4, 5] μC/cm^2a^	1.44 (0.76–2.72)	0.99	0.51 (0.30–0.87)	0.17
Charge density (5, 6] μC/cm^2a^	1.31 (0.67–2.56)	0.99	0.82 (0.47–1.42)	0.99
Charge density (6, 7] μC/cm^2a^	0.43 (0.03–5.55)	0.99	0.82 (0.08–8.92)	0.99
Charge density >7 μC/cm^2a^	3.29 (0.07–157.24)	0.99	0.25 (0.01–4.97)	0.99
Frequency (0, 20 Hz]^b^	1.04 (0.45–2.42)	0.99	1.81 (0.72–4.55)	0.99
Frequency (20, 100 Hz)^b^	**3.80** (**1.70–8.51)**	**0**.**02**	**0.07** (**0.03–0.17)**	**<0**.**001**
Frequency 100 Hz^b^	2.01 (1.15–3.52)	0.20	**0.23** (**0.13–0.43)**	**<0**.**001**
Frequency (100, 200 Hz)^b^	2.04 (0.85–4.91)	0.99	1.03 (0.40–2.66)	0.99
Frequency >200 Hz^b^	1.38 (0.59–3.22)	0.99	1.30 (0.52–3.24)	0.99
Pulse width <160 μs^c^	1.18 (0.89–1.57)	0.99	1.14 (0.88–1.47)	0.99
Pulse width >160 μs^c^	1.19 (0.98–1.43)	0.90	0.84 (0.69–1.01)	0.76
Burst duration <100 ms^d^	Not trialed			
Burst duration (100, 500] ms^d^	1.06 (0.80–1.40)	0.99	0.89 (0.67–1.19)	0.99
Burst duration >500 ms^d^	**0.41** (**0.32–0.52)**	**<0**.**001**	**2.18** (**1.69–2.83)**	**<0**.**001**
Number of detection events that triggered a therapy	0.93 (0.90–0.97)	<0.001	1.25 (1.21–1.29)	<0.001
Days since implant	0.92 (0.65–1.32)	0.99	1.40 (0.96–2.03)	0.84

Neocortical leads (*n* = 31)				
	Low-risk state (R^2^_marginal_ = 0.04, R^2^_conditional_ = 0.31, AIC 101 212.7)		High-risk state (R^2^_marginal_ = 0.01, R^2^_conditional_ = 0.48, AIC 100 305.7)	
	Coefficient estimate (SE)	Adjusted *P*-value	Coefficient estimate (SE)	Adjusted *P*-value
Intercept	35.44 (23.23–54.07)	<0.001	0.05 (0.02–0.09)	<0.001
Charge density (0.5, 3] μC/cm^2a^	1.14 (1.01–1.28)	0.31	1.06 (0.95–1.19)	0.99
Charge density (3, 4] μC/cm^2a^	0.80 (0.67–0.94)	0.11	**1.44** (**1.22–1.70)**	**0**.**003**
Charge density (4, 5] μC/cm^2a^	0.91 (0.77–1.09)	0.99	**1.31** (**1.10–1.56)**	**0**.**03**
Charge density (5, 6] μC/cm^2a^	0.87 (0.70–1.07)	0.99	**1.38** (**1.11–1.71)**	**0**.**04**
Charge density (6, 7] μC/cm^2a^	1.26 (1.03–1.55)	0.30	1.28 (1.05–1.56)	0.12
Charge density >7 μC/cm^2a^	1.24 (0.97–1.57)	0.80	1.34 (1.06–1.70)	0.12
Frequency (0, 20 Hz]^b^	0.82 (0.72–0.94)	0.06	**1.37** (**1.20–1.56)**	**<0**.**001**
Frequency (20, 100 Hz)^b^	1.00 (0.85–1.18)	0.99	1.05 (0.89–1.24)	0.99
Frequency 100 Hz^b^	1.09 (0.96–1.24)	0.99	1.18 (1.04–1.34)	0.10
Frequency (100, 200 Hz)^b^	1.07 (0.93–1.24)	0.99	**1.24** (**1.07–1.43)**	**0**.**04**
Frequency >200 Hz^b^	**4.78** (**2.10–10.89)**	**0**.**003**	1.05 (0.14–7.87)	0.99
Pulse width <160 μs^c^	1.08 (0.95–1.22)	0.99	0.93 (0.82–1.05)	0.99
Pulse width >160 μs^c^	1.08 (0.97–1.20)	0.99	1.02 (0.91–1.13)	0.99
Burst duration <100 ms^d^	0.96 (0.83–1.10)	0.99	1.20 (1.04–1.38)	0.11
Burst duration (100, 500] ms^d^	0.90 (0.82–0.98)	0.22	**1.28** (**1.17–1.40)**	**<0**.**001**
Burst duration >500 ms^d^	**0.48** (**0.39–0.60)**	**<0**.**001**	**0.55** (**0.45–0.67)**	**<0**.**001**
Number of detection events that triggered a therapy	0.82 (0.80–0.84)	<0.001	1.01 (0.99–1.03)	0.99
Days since implant	1.08 (0.88–1.34)	0.99	1.09 (0.88–1.33)	0.99

Fixed effects for stimulation parameters on probability of remaining in or transitioning to a low-risk state are shown. Associations are based on values of charge density, stimulation frequency, pulse width and burst duration averaged across the first two therapy bursts. Adjusted *P*-values after Holm correction are shown. Bold values indicate those significant at the 0.05 level after family-wise error rate control. AIC = Akaike information criterion; SE = standard error.

^a^Relative to charge density of ≤0.5 μC/cm^2^.

^b^Relative to stimulation frequency of 200 Hz.

^c^Relative to pulse width of 160 μs.

^d^Relative to burst duration of 100 ms.

### Monopolar neocortical stimulation

**Figure 3:**
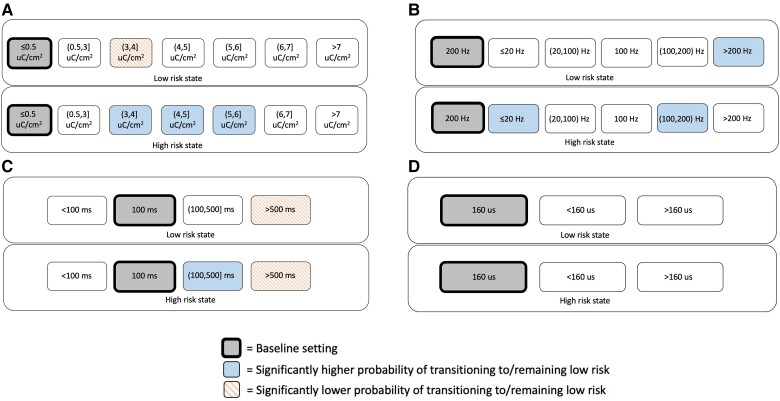
**For monopolar neocortical stimulation, associations of brain-responsive neurostimulation (RNS) using RNS System stimulation parameter settings with the probability of transitioning to or remaining in a low-risk state, conditional on current state.** (**A**) Charge density, (**B**) stimulation frequency, (**C**) burst duration and (**D**) pulse width. Baseline setting is shown in grey (bolded rectangles). Blue solid shading indicates settings found to have a significantly higher probability of transitioning to or remaining in a low-risk state relative to the baseline setting. Orange dashed shading indicates settings found to have a significantly lower probability of transitioning to or remaining in a low-risk state relative to the baseline setting. Parameter ranges over the two bursts in the first therapy: charge densities of 0.30–24.10 μC/cm^2^, stimulation frequencies of 1–333.3 Hz, burst durations of 10–5000 ms and pulse widths of 80–760 µs.

Thirty-one patients with neocortical leads underwent monopolar or lead-to-lead stimulation during RNS therapy. Table 3 shows the association of RNS System stimulation parameters with the probability of remaining/transitioning to a low-risk state in monopolar neocortical stimulation. For monopolar neocortical stimulation, the use of high charge densities above 3 μC/cm^2^ was associated with a greater probability of transitioning to a low-risk state for times when patients were in high-risk states, with less of a clear benefit when patients were in low-risk states (Table 3 and Fig. 3A). High frequency stimulation (200–333.3 Hz) was most effective in low-risk states and significantly associated with a higher probability of remaining low-risk (*P* < 0.001), whereas low frequency stimulation (1–20 Hz) was most effective in high-risk states and significantly associated with a higher probability of transitioning to a low-risk state (*P* < 0.001; Fig. 3B). Long burst durations (100–500 ms) were associated with a transition to a low-risk state when applied during a high-risk state, but with a statistically non-significant transition to a high-risk state when applied during a low-risk state. Very long (500–5000 ms) burst durations were associated with a lower chance of being low-risk in the subsequent hour if applied either in high or low-risk states (*P* < 0.001 and *P* < 0.001; Fig. 3C). There was no significant difference between 160 μs, <160 μs and >160 μs pulse widths in association with subsequent transitions to low-risk states (Fig. 3D). Overall, responsive neurostimulation using monopolar neocortical stimulation explained 4% of the variance in hourly state transitions during low-risk states and 1% during high-risk states, corresponding to a small effect size.

### Bipolar mesiotemporal stimulation

**Figure 4:**
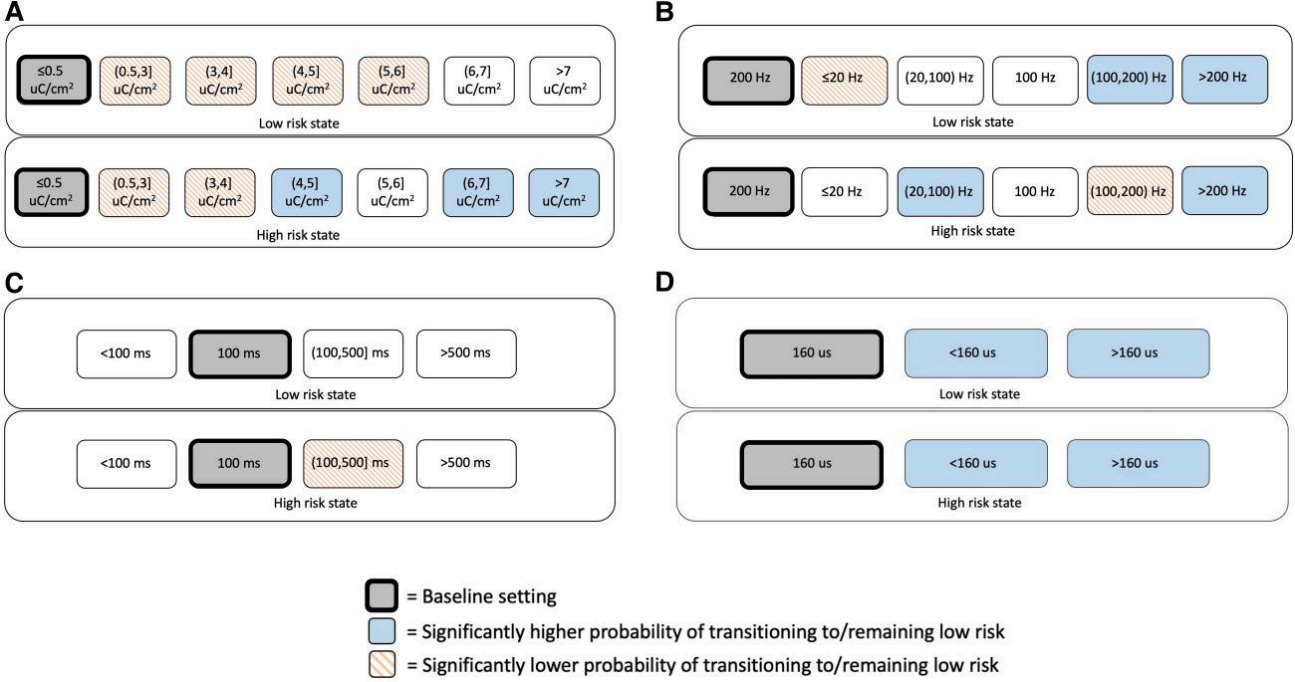
**For bipolar mesial temporal stimulation, associations of brain-responsive neurostimulation (RNS) using RNS System stimulation parameter settings with the probability of transitioning to or remaining in a low-risk state, conditional on current state.** (**A**) Charge density, (**B**) stimulation frequency, (**C**) burst duration and (**D**) pulse width. Baseline setting is shown in grey (bolded rectangles). Blue solid shading indicates settings found to have a significantly higher probability of transitioning to or remaining in a low-risk state relative to the baseline setting. Orange dashed shading indicates settings found to have a significantly lower probability of transitioning to or remaining in a low-risk state relative to the baseline setting. Average parameter ranges over the two bursts in the first therapy were charge densities of 0.40–8.90 μC/cm^2^, stimulation frequencies of 2–333.3 Hz, burst durations of 10–5000 ms and pulse widths of 80–400 µs.

Forty-five patients with mesiotemporal leads underwent bipolar stimulation during RNS therapy. Table 4 shows the association of RNS System stimulation parameters with the probability of remaining/transitioning to a low-risk state using bipolar mesiotemporal stimulation. Use of high charge densities above 3 μC/cm^2^ was generally more beneficial in high- than low-risk states (Table 4 and Fig. 4A). When applied during low-risk states, higher stimulation frequencies (>100 to <200 Hz and 200 to 333.3 Hz) were associated with a higher probability of remaining low-risk than 200 Hz stimulation, while during high-risk states, either low frequency stimulation (>20 to <100 Hz) or very high frequency stimulation (200 to 333.3 Hz) was associated with a higher probability of transitioning to a low-risk state compared to 200 Hz stimulation (Table 4 and Fig. 4B). In high-risk states, long burst durations (100 to 500 ms) were associated with a lower probability of transitioning to a low-risk state compared to a burst duration of 100 ms (*P* < 0.001; Fig. 4C). Pulse widths of either less than or greater than 160 μs were associated with a greater probability of subsequently being low-risk compared to a pulse width of 160 μs, regardless of whether used in a high- or low-risk state (*P* < 0.001; Fig. 4D). Overall, responsive neurostimulation using monopolar mesiotemporal stimulation explained 1% of the variance in hourly state transitions for both low-risk and high-risk states, corresponding to a small effect size.

**Table 4:** Mesial temporal versus neocortical state-dependent parameter associations: bipolar stimulation

Mesial temporal leads (*n* = 45)				
	Low-risk state (R^2^_marginal_ = 0.01, R^2^_conditional_ = 0.41, AIC 186 688.3)		High-risk state (R^2^_marginal_ = 0.01, R^2^_conditional_ = 0.47, AIC 193 479.5)	
	Coefficient estimate (SE)	Adjusted *P*-value	Coefficient estimate (SE)	Adjusted *P*-value
Intercept	41.52 (27.36–63.00)	<0.001	0.05 (0.03–0.09)	<0.001
Charge density (0.5, 3] μC/cm^2a^	**0.86** (**0.79–0.94)**	**0**.**01**	**0.89** (**0.82–0.97)**	**0**.**06**
Charge density (3, 4] μC/cm^2a^	**0.63** (**0.56–0.71)**	**<0**.**001**	**0.74** (**0.66–0.83)**	**<0**.**001**
Charge density (4, 5] μC/cm^2a^	**0.83** (**0.74–0.94)**	**0**.**02**	**1.18** (**1.05–1.32)**	**0**.**04**
Charge density (5, 6] μC/cm^2a^	**0.77** (**0.68–0.88)**	**0**.**002**	1.05 (0.92–1.20)	0.99
Charge density (6, 7] μC/cm^2a^	0.88 (0.76–1.02)	0.50	**1.61** (**1.40–1.85)**	**<0**.**001**
Charge density >7 μC/cm^2a^	1.02 (0.87–1.19)	0.99	**1.54** (**1.32–1.80)**	**<0**.**001**
Frequency (0.20 Hz]^b^	**0.63** (**0.55–0.71)**	**<0**.**001**	0.97 (0.85–1.09)	0.99
Frequency (20 100 Hz)^b^	1.16 (0.94–1.42)	0.80	**1.44** (**1.18–1.77)**	**0**.**004**
Frequency 100 Hz^b^	0.99 (0.89–1.11)	0.99	0.99 (0.88–1.10)	0.99
Frequency (100, 200 Hz)^b^	**1.38** (**1.27–1.50)**	**<0**.**001**	**0.77** (**0.72–0.84)**	**<0**.**001**
Frequency >200 Hz^b^	**1.13** (**1.05–1.21)**	**0**.**008**	**1.28** (**1.19–1.37)**	**<0**.**001**
Pulse width <160 μs^c^	**1.18** (**1.09–1.28)**	**<0**.**001**	**1.47** (**1.36–1.60)**	**<0**.**001**
Pulse width > 160 μs^c^	**1.14** (**1.06–1.21)**	**0**.**002**	**1.16** (**1.09–1.24)**	**<0**.**001**
Burst duration <100 ms^d^	0.93 (0.81–1.08)	0.99	1.18 (1.01–1.36)	019
Burst duration (100 500] ms^d^	1.08 (1.00–1.16)	0.36	**0.84** (**0.78–0.91)**	**<0**.**001**
Burst duration >500 ms^d^	2.16 (0.99–4.71)	0.37	0.45 (0.21–0.97)	0.21
Number of detection events that triggered a therapy	0.88 (0.87–0.90)	<0.001	0.97 (0.96–0.99)	0.004
Days since implant	1.03 (0.83–1.27)	0.99	1.05 (0.96–1.14)	0.99

Neocortical leads (*n* = 7)				
	Low-risk state (R^2^_marginal_ = 0.26, R^2^_conditional_ = 0.26, AIC 12 700)		High-risk state (R^2^_marginal_ = 0.32, R^2^_conditional_ = 0.32, AIC 12 010)	
	Coefficient estimate (SE)	Adjusted *P*-value	Coefficient estimate (SE)	Adjusted *P*-value
Intercept	15.45 (14.35–16.73)	<0.001	0.04 (0.04–0.04)	<0.001
Charge density^e^	**1.23** (**1.12–1.39)**	**<0**.**001**	**0.48** (**0.42–0.55)**	**<0**.**001**
Frequency^e^	**1.63** (**1.41–1.92)**	**<0**.**001**	**0.62** (**0.55–0.69)**	**<0**.**001**
Burst duration^e^	**3.01** (**2.46–3.74)**	**<0**.**001**	**0.62** (**0.52–0.74)**	**<0**.**001**
Pulse width^e^	**1.11** (**1.06–1.16)**	**<0**.**001**	Not estimated^f^	**–**
Number of detection events that triggered a therapy	1.27 (1.17–1.39)	<0.001	2.55 (2.45–2.67)	<0.001
Days since implant	1.03 (0.97–1.10)	0.34	1.20 (1.13–1.27)	<0.001

Fixed effects for stimulation parameters on probability of remaining or transitioning to a low-risk state are shown. Associations are based on values of charge density, stimulation frequency, pulse width and burst duration averaged across the first two therapy bursts. Adjusted *P*-values after Holm correction are shown. Bold values indicate those significant at the 0.05 level with family-wise error rate control. AIC = Akaike information criterion; SE = standard error.

^a^Relative to charge density of ≤0.5 μC/cm^2^.

^b^Relative to stimulation frequency of 200 Hz.

^c^Relative to pulse width of 160 μs.

^d^Relative to burst duration of 100 ms.

^e^Using original values due to limited variation in values for categorization.

^f^Not estimated due to singularity.

### Bipolar neocortical stimulation

Bipolar stimulation of neocortical seizure foci was used in seven patients meeting inclusion criteria. Higher charge densities, higher stimulation frequencies, longer burst durations and longer pulse widths were associated with a greater chance of remaining low-risk when applied in low-risk states (*P* < 0.001), whereas lower charge densities, lower stimulation frequencies and shorter burst durations were associated with a greater chance of transitioning to a low-risk state if applied during high-risk states (*P* < 0.001) (Table 4). For times identified to be high-risk states, only 2 h employed a pulse width other than 160 μs (2 h with pulse width of 120 μs; 30 368 h with pulse width of 160 μs); therefore, the effect of pulse width was not analysed for neocortical bipolar stimulation during high-risk states. Overall, responsive neurostimulation using monopolar mesiotemporal stimulation explained 26% of the variance in hourly state transitions during low-risk states and 32% during high-risk states, corresponding to a large effect size.

## Discussion

Here, leveraging a unique dataset of patients treated with the RNS System in a large, multi-centre clinical trial, we performed a retrospective statistical analysis to test a hypothesis that the acute effects of responsive neurostimulation on seizure risk depend on the timing and location of stimulation. We found that probabilities of transitions between states of different seizure risk depend on the stimulation parameters being changed, the starting seizure risk state and the stimulated brain region. Thus, the acute therapeutic effects of responsive neurostimulation appear to be a function of how, when and where stimulation is applied.

There are several main contributions of this study. First, our findings suggest that optimal stimulation parameters for acute impact on seizure risk may differ between neocortical and mesiotemporal epilepsies. Typical recommendations are to start with monopolar or lead-to-lead stimulation for neocortical seizure foci and with bipolar stimulation for mesiotemporal seizure foci. Based on our results, divergence between these treatment algorithms should be expanded to include other stimulation parameters, including charge density and stimulation frequency, for which changes had opposing effects on risk state transitions depending on seizure localization.

Second, we confirm and extend previous findings on state-dependent effects of stimulation parameter changes.^11^ The fundamental principle proposed previously^11^ and confirmed here in a larger independent study—that effects of stimulation parameter changes appear to depend on current brain state and location of stimulation—has practical implications for clinicians using the RNS System. For example, in a patient with mesiotemporal epilepsy treated with monopolar stimulation, the parameter changes most likely to be favourable depend on whether the patient is doing well (low-risk state) or poorly (high-risk state): lower stimulation frequencies (20–100 Hz) may be favoured in the former scenario, whereas longer burst durations (>500 ms) may be favoured in the latter. Recent work suggests that responsive neurostimulation during low-risk states (i.e. periods of time with fewer than usual LEs) may be more effective^16^; it would be of interest to examine whether stimulation parameters used during these low-risk states differed systematically from those used during high-risk states. This work advances findings from our previous single-centre study by showing that the same phenomenon generalizes to a larger, independent, multi-centre cohort, increasing the likelihood of reproducibility and laying the groundwork for future prospective studies.

Third, whereas previous studies investigating the relationship between responsive neurostimulation and time-varying changes in risk state have defined risk states based only on LEs,^11,16^ this study shows that consideration of the phase of circadian and multidien IEA cycles during which stimulation is applied may result in different effects on brain state.^30^ Low- and high-risk states were identified using dimension reduction techniques that simultaneously analysed the phase of IEA cycles along with LE counts; changes in stimulation parameters produced different outcomes depending on the state when changes were made. Phase-dependent stimulation effects on plasticity have been shown in rodents, with stimulation inducing long-term potentiation differentially depending on the phase of theta oscillations when stimulation is applied.^31^ In humans, IEA cycle phase has demonstrated value for forecasting seizures^13,32-35^ and for increasing the yield of diagnostic monitoring,^36^ but its utility for optimizing patterns of therapeutic brain stimulation has not been explored. Our results suggest that the brain may respond differently to electrical stimulation at different times during IEA cycles, and the underlying mechanisms warrant further investigation. Recent work has shown that seizures unfold through a sequence of network states that depends on IEA cycle phase.^37,38^ If seizures are ‘built’ differently at different times of IEA cycles, the most effective way to ‘dismantle’ them with neurostimulation might vary accordingly.

Fourth, this study is consistent with emerging evidence that there is a baseline rate of transition between seizure risk states,^14,17-19^ which can be affected either acutely or in the long run by RNS System treatment; indeed, there is growing awareness that acute effects of responsive neurostimulation may be paralleled by effects that unfold over long periods of time.^3,6,21,39,40^ The magnitude of the regression intercepts for acute risk state transitions (which reflects the baseline rate of transitions in the absence of stimulation), relative to the magnitude of the coefficients for RNS System stimulation parameters, suggests a strong baseline effect of hourly state transitions, which is partially modulated in the acute setting by responsive neurostimulation. Prior work has demonstrated a strong effect for a baseline rate of transition between seizure risk states in the absence of RNS^17-19^; the current work directly quantifies the impact of responsive neurostimulation on this transition rate. For neocortical stimulation, we found a small effect size for marginal R^2^-values using monopolar stimulation and a large effect size for marginal R^2^-values using bipolar stimulation, indicating that responsive neurostimulation explains a limited portion of variance in acute seizure risk state transitions when using monopolar stimulation and a greater portion of variance in acute risk state transitions when using bipolar stimulation. Conversely, for mesiotemporal stimulation, we found a small effect size for marginal R^2^-values using bipolar stimulation and a medium effect size for marginal R^2^-values using monopolar stimulation in high-risk states, indicating that responsive neurostimulation may exert its greatest potential impact on acute risk state modulation via monopolar stimulation during high-risk states. Clinically, one practical implication of this finding is that certain stimulation montages, such as bipolar stimulation for neocortical epilepsies or monopolar stimulation for mesiotemporal epilepsies, may be more amenable to acute effects on seizure risk state modulation,^21^ whereas other stimulation montages, such as monopolar stimulation for neocortical epilepsies or bipolar stimulation for mesiotemporal epilepsies, may be less amenable to acute effects, possibly favouring instead the chronic neuromodulatory effects on seizure networks that have been demonstrated in other studies.^6,41^ A second practical implication is that the occurrence of seizures soon after stimulation parameters are changed should be considered to be potentially due to baseline transition rates rather than to changes in stimulation parameters.

Last, the large and diverse cohort studied here supports generalizability of the principle of state-dependent effects of neurostimulation. Epilepsy is conceptualized as a disorder of brain networks,^42^ which are dynamic over diverse timescales,^37^ and optimal therapies for reducing seizures might need to mirror this dynamism. Clinical outcomes currently observed with the RNS System may reflect the average of ‘productive’ and ‘counterproductive’ effects of the relatively invariant stimulation parameters employed in current paradigms. State-dependent effects of neurostimulation may also help explain the similarity in outcomes between open-loop (i.e. scheduled intermittent stimulation non-specific to seizure risk state) deep brain stimulation and closed-loop responsive neurostimulation for epilepsy,^43,44^ though head-to-head comparative data are lacking. The picture now emerging is that effective RNS therapy depends less on patient clinical characteristics and more on stimulation of a conducive network substrate,^7,8^ in the right place^9^ and at the right time^11^ in order to engage long-term network plasticity.^6,40,41^ This framework motivates development of next-generation devices capable of sensing momentary network state and delivering adaptive, risk-stratified, localization-specific stimulation to maximize therapeutic effects.

Mechanisms underlying the observed state- and location-dependence of responsive neurostimulation effects remain unclear. Recent evidence that background EEG features fluctuate in relation to multidien IEA cycle phase^45^ suggests that brain network properties vary in relation to seizure risk. Thus, a speculative possibility is that the state-dependence of stimulation effects stems from the dynamism of the network substrate itself. Similarly, given fundamental differences in cellular composition and functional connectivity between the neocortex and mesial temporal lobes, it is likely that the electrical stimulation parameters necessary to synchronize, desynchronize, excite or inhibit cells may differ based on stimulation location.^46^ Furthermore, recent work has revealed that epileptic networks may have critical nodes, anatomic sites defined by patterns of connectivity, where responsive neurostimulation is most effective for reducing seizures,^10^ and these points of susceptibility to focal stimulation likely differ between neocortical and mesial temporal locations.

This study has limitations. First, only 1-h risk look-ahead horizons for risk-state transitions were evaluated; longer horizons will be investigated in future research. Second, the stimulation parameter space was not uniformly sampled, and due to the retrospective nature of this study, there may be co-dependencies of stimulation parameter values; for example, low stimulation frequencies are likely to be paired with long burst durations, as it would not make sense to pair low stimulation frequencies with short burst durations. Third, this study investigates only the main effects of stimulation parameters, as the sample size is not powered to infer interaction effects; prospective studies on targeted parameter combinations will be needed to parse interactions between stimulation parameters (such as the dependence of charge density on stimulation frequency). Fourth, although the temporal directionality relating stimulation parameter values to future changes in risk allows for causal inference, prospective testing is ultimately needed to confirm cause-and-effect relationships. Finally, in this study, marginal R^2^-values provide a measure of the variability in risk state transitions explained by fixed effects (here, RNS System stimulation parameter changes) rather than seizure frequency reduction, which limits direct clinical translatability and suggests an avenue for future research. This study demonstrates the generalizability of a previously preliminary hypothesis of state-dependent neurostimulation effects in a large, multi-centre cohort, pointing to the need for a prospective study as a next step towards the development of adaptive neurostimulation devices.

## Supplementary Material

awae240_Supplementary_Data

## Data Availability

Study data are proprietary to NeuroPace, Inc. and are not available except via a negotiated Data Use and Confidentiality Agreement.
