# Supplementary material for: State-dependent effects of responsive neurostimulation depend on seizure localization
Source: Brain. 2024 Jul 25;148(2):521–32. doi: 10.1093/brain/awae240 (PMC11788193; doi:10.1093/brain/awae240)
Supplement: awae240_Supplementary_Data [file awae240_supplementary_data.pdf]

Supplementary Materials

Supplementary Table S1. Stimulation configurations used in first therapy. Number of patient-hours and sample size for each stimulation configuration are shown. Grouped bipolar and mixed configurations were excluded from analysis due to low sample size. †Parentheses indicate anode (+) and cathode (-) designations for four-electrode RNS System leads during Burst 1 and Burst 2 of the first delivered therapy. If only (+) or (-) are shown, the neurostimulator canister is of the opposite polarity.

	Burst 1 (lead 1)(lead 2) Burst 2 (lead 1)(lead 2)†	Number of patient-hours	Sample size
Monopolar and/or lead-to-lead in bursts 1 and 2	(+0+0)(0-0-) (0+0+)(-0-0)<br>(----)(0000) (0000)(----)<br>(-0-0)(0-0-) (0-0-)(-0-0)<br>(++++)(----) (0000)(0000)<br>(0000)(++++) (++++)(0000)<br>(++++)(----) (----)(++++)<br>(+++0)(----) (0000)(0000)<br>(+0+0)(0000) (0000)(0000)<br>(+++0)(----) (+++0)(----)<br>(+0+0)(0000) (0+00)(0000)<br>(---0)(0000) (0000)(----)<br>(++00)(0000) (0000)(++++)<br>(+000)(+0+0) (0+00)(0+0+)<br>(---0)(----) (--00)(--00)<br>(++++)(0000) (0000)(++++)<br>(00++)(00++) (++00)(++00)<br>(----)(0000) (0000)(-0-0)<br>(0+0+)(-0-0) (+0+0)(0-0-)<br>(++++)(0000) (0000)(++00)<br>(++++)(----) (++++)(----)<br>(0+0+)(0000) (+0+0)(0000)<br>(----)(++++) (0000)(0000)<br>(--00)(++00) (00--)(00++)<br>(----)(++++) (----)(++++)<br>(++00)(----) (0000)(0000)<br>(--00)(++++) (0000)(00--)<br>(--00)(++++) (0000)(0000)<br>(-000)(++++) (0000)(0000)<br>(0000)(----) (----)(0000)<br>(--00)(--00) (0000)(0000)<br>(----)(++++) (++++)(----)<br>(--00)(0000) (0000)(--00)<br>(0000)(++++) (0000)(0000)<br>(----)(0000) (0000)(0000)<br>(0000)(00++) (00++)(0000)<br>(0000)(----) (++++)(0000)	864,430	52

	(++00)(--00) (00++)(00--)<br>(0000)(-0-0) (0000)(0-0-)<br>(++++)(++++) (++++)(++++)<br>(0000)(+++0) (0000)(----)<br>(++++)(----) (0000)(----)<br>(--00)(--00) (++00)(++00)<br>(++00)(++00) (++00)(++00)<br>(++00)(0000) (0000)(++00)<br>(----)(----) (0000)(0000)<br>(+0+0)(0+0+) (0+0+)(+0+0)<br>(----)(0000) (----)(----)<br>(++++)(0000) (++++)(0000)<br>(++++)(0000) (0000)(0000)<br>(++++)(++++) (0000)(0000)<br>(+++0)(---0) (---0)(+++0)<br>(0000)(----) (0000)(----)<br>(++++)(00++) (0000)(00++)<br>(0000)(0---) (++++)(0000)<br>(0+++)(0---) (0---)(0+++)<br>(----)(----) (----)(----)<br>(++++)(++++) (----)(----)<br>(0000)(00--) (----)(0000)		
Bipolar in bursts 1 and 2	(-+-+)(-+-+) (0000)(0000)<br>(-00+)(-00+) (0000)(0000)<br>(+00-)(+00-) (0000)(0000)<br>(0000)(+00-) (+00-)(0000)<br>(00-+)(-+00) (-+00)(00-+)<br>(+-++)(0000) (0000)(++0-)<br>(++00)(+---) (0000)(0000)<br>(+-00)(0000) (0000)(+-00)<br>(-+-+)(0000) (0000)(+-+-)<br>(0000)(-+00) (-+00)(0000)<br>(+-+-)(+-+-) (0000)(0000)<br>(000+)(+-+-) (+-+-)(0000)<br>(+-+-)(0000) (0000)(+-+-)<br>(+-+-)(+-+-) (+-+-)(+-+-)<br>(+0-0)(0000) (0000)(+--0)<br>(-+00)(0000) (0000)(-+00)<br>(+-00)(0000) (0-+0)(0000)<br>(00-+)(0000) (0000)(00-+)	1,350,148	52

	(+-00)(+-00) (00+-)(00+-)<br>(0000)(+-+-) (+-+-)(0000)<br>(0000)(+-00) (+-00)(0000)<br>(+-00)(+-00) (0000)(0000)<br>(+-+-)(0000) (0000)(0000)<br>(+-+-)(++++) (0000)(0000)<br>(+-+-)(0000) (+-+-)(0000)<br>(0000)(+-+-) (0000)(0000)<br>(0000)(00+-) (0000)(00-+)<br>(0000)(00+-) (+-+-)(0000)<br>(0000)(+-+-) (0000)(+-+-)<br>(+-+-)(++++) (++++)(+-+-)<br>(+-+-)(----) (----)(--+-)<br>(+-+0)(+-+0) (-+-0)(-+-0)<br>(+-+-)(+-+-) (-+-+)(-+-+)<br>(0000)(-+-+) (-+-+)(0000)<br>(-0+0)(0000) (0-0+)(0000)<br>(-+-+)(0000) (0000)(0000)<br>(0000)(+-+-) (0000)(-+-+)<br>(0000)(-+-+) (-+00)(0000)<br>(0000)(-+-+) (0000)(-+-+)<br>(0000)(-+-+) (+-+-)(0000)<br>(-+-+)(0000) (0000)(-+-+)<br>(+-0+)(0000) (-+0-)(0000)<br>(+-0+)(0000) (0000)(0000)<br>(+-0+)(0000) (0000)(+-0+)		

Supplementary Table S2. Estimated standard deviation of random effects for generalized linear mixed effects models.

**(A) Mesial temporal leads, monopolar stimulation**					
		**Low-risk state**		**High-risk state**	
Group	Random Effect	Standard Deviation	Correlation	Standard Deviation	Correlation
Patient	Intercept	1.17		1.19	
	Time since implant, in days	0.51	-0.63	0.56	0.01
**(B) Neocortical leads, monopolar stimulation**					
		**Low-risk state**		**High-risk state**	
Group	Random Effect	Standard Deviation	Correlation	Standard Deviation	Correlation
Patient	Intercept	1.03		1.67	
	Time since implant, in days	0.46	-0.03	0.38	0.37
**(C) Mesial temporal leads, bipolar stimulation**					
		**Low-risk state**		**High-risk state**	
Group	Random Effect	Standard Deviation	Correlation	Standard Deviation	Correlation
Patient	Intercept	1.35		1.66	
	Time since implant, in days	0.66	0.32	0.22	-0.05

Supplementary Table S3. Univariate associations between RNS stimulation parameter categories and transition to a low-risk state: Monopolar stimulation of mesial temporal leads. Percentage of time (0-1) that a stimulation parameter was followed by a low-risk state in the subsequent hour. Baseline category is shaded in gray.

(A) Charge density

	Applied in low risk state	Applied in high risk state
≤0.5 μC/cm2	0.916 (7180 patient-hours, n=5)	0.059 (10135 patient-hours, n=5)
(0.5, 3] μC/cm2	0.965 (89882 patient-hours, n=17)	0.049 (63275 patient-hours, n=17)
(3, 4] μC/cm2	0.947 (17747 patient-hours, n=4)	0.084 (11171 patient-hours, n=5)
(4, 5] μC/cm2	0.971 (3444 patient-hours, n=2)	0.035 (2818 patient-hours, n=2)
(5, 6] μC/cm2	0.976 (9021 patient-hours, n=2)	0.029 (7609 patient-hours, n=2)
(6, 7] μC/cm2	0.974 (739 patient-hours, n=2)	0.041 (461 patient-hours, n=2)
>7 μC/cm2	0.993 (3206 patient-hours, n=1)	0.008 (2453 patient-hours, n=1)

(B) Frequency

	Applied in low risk state	Applied in high risk state
(0,20 Hz]	0.939 (16080 patient-hours, n=2)	0.082 (9604 patient-hours, n=2)
(20,100 Hz)	0.970 (10490 patient-hours, n=1)	0.046 (6846 patient-hours, n=1)
100 Hz	0.962 (7791 patient-hours, n=3)	0.040 (7335 patient-hours, n=3)
(100, 200 Hz)	0.972 (39240 patient-hours, n=4)	0.076 (14325 patient-hours, n=4)
200 Hz	0.955 (35990 patient-hours, n=13)	0.041 (39815 patient-hours, n=13)
> 200 Hz	0.967 (21628 patient-hours, n=4)	0.047 (15021 patient-hours, n=4)

(C) Pulse width

	Applied in low risk state	Applied in high risk state
<160 ms	0.966 (29172 patient-hours, n=8)	0.059 (30515, n=9)
160 ms	0.965 (62495 patient-hours, n=11)	0.048 (46310, n=11)
>160 ms	0.954 (39551 patient-hours, n=9)	0.059 (30515, n=9)

(D) Burst duration

	Applied in low risk state	Applied in high risk state
<100 ms	Not trialed	Not trialed
100 ms	0.967 (103928 patient-hours, n=17)	0.053 (65426 patient-hours, n=17)
(100,500] ms	0.943 (25176 patient-hours, n=6)	0.045 (31610 patient-hours, n=6)
>500 ms	0.938 (2114 patient-hours, n=1)	0.148 (886 patient-hours, n=1)

Supplementary Table S4. Univariate associations between RNS stimulation parameter categories and transition to a low-risk state: Monopolar stimulation of neocortical leads. Percentage of time that a stimulation parameter was followed by a low-risk state in the subsequent hour. Baseline category is shaded in gray.

(A) Charge density

	Applied in low risk state	Applied in high risk state
≤0.5 μC/cm2	0.871 (T=7772, n=3)	0.060 (T=16614, n=3)
(0.5, 3] μC/cm2	0.961 (T=149753, n=23)	0.041 (T=140953, n=24)
(3, 4] μC/cm2	0.972 (T=47331, n=13)	0.067 (T=19287, n=13)
(4, 5] μC/cm2	0.974 (T=62915, n=14)	0.055 (T=29812, n=14)
(5, 6] μC/cm2	0.922 (T=6167, n=5)	0.078 (T=6144, n=5)
(6, 7] μC/cm2	0.951 (T=50106, n=10)	0.056, (T=43982, n=10)
>7 μC/cm2	0.948 (T=13285, n=5)	0.063 (T=10863, n=5)

(B) Frequency

	Applied in low risk state	Applied in high risk state
(0,20 Hz]	0.962 (T=74691, n=10)	0.042 (T=6606, n=10)
(20,100 Hz)	0.946 (T=122225, n=19)	0.056 (T=118283, n=19)
100 Hz	0.946 (T=108621, n=17)	0.057 (T=103225, n=17)
(100, 200 Hz)	0.972 (T=67511, n=10)	0.043 (T=44012, n=10)
200 Hz	0.963 (T=55950, n=16)	0.054 (T=37990, n=16)
> 200 Hz	0.997 (T=16952, n=2)	0.034 (T=1364, n=2)

(C) Pulse width

	Applied in low risk state	Applied in high risk state
<160 ms	0.953 (T=47178, n=11)	0.056 (T=39411, n=12)
160 ms	0.962 (T=228160, n=28)	0.047 (T=184731, n=28)
>160 ms	0.960 (T=61991, n=8)	0.057 (T=43513, n=8)

(D) Burst duration

	Applied in low risk state	Applied in high risk state
<100 ms	0.968 (T=55122, n=4)	0.045 (T=38697, n=4)
100 ms	0.961 (T=223919, n=29)	0.047 (T=182385, n=30)
(100,500] ms	0.951 (T=53196, n=11)	0.071 (T=36577, n=11)
>500 ms	0.924 (T=5092, n=3)	0.039 (T=9996, n=3)

Supplementary Table S5. Univariate associations between RNS stimulation parameter categories and transition to a low-risk state: Bipolar stimulation of mesial temporal leads. Percentage of time that a stimulation parameter was followed by a low-risk state in the subsequent hour. Baseline category is shaded in gray.

(A) Charge density

	Applied in low risk state	Applied in high risk state
≤0.5 μC/cm2	0.888 (T=30522, n=6)	0.071 (T=48434, n=6)
(0.5, 3] μC/cm2	0.968 (T=314704, n=35)	0.039 (T=256269, n=35)
(3, 4] μC/cm2	0.949 (T=86817, n=17)	0.038 (T=115847, n=19)
(4, 5] μC/cm2	0.976 (T=133100, n=20)	0.049 (T=65638, n=21)
(5, 6] μC/cm2	0.978 (T=79042, n=12)	0.032 (T=55621, n=11)
(6, 7] μC/cm2	0.950 (T=27636, n=11)	0.047 (T=29036, n=11)
>7 μC/cm2	0.947 (T=16958, n=5)	0.063 (T=14157, n=5)

(B) Frequency

	Applied in low risk state	Applied in high risk state
(0,20 Hz]	0.868 (T=18419, n=3)	0.072 (T=33759, n=3)
(20,100 Hz)	0.901 (T=1253, n=1)	0.100 (T=1242, n=1)
100 Hz	0.967 (T=59104, n=8)	0.040 (T=48268, n=8)
(100, 200 Hz)	0.970 (T=110586, n=17)	0.033 (T=98174, n=17)
200 Hz	0.965 (T=431911, n=39)	0.044 (T=342931, n=39)
> 200 Hz	0.965 (T=67506, n=18)	0.038 (T=60628, n=18)

(C) Pulse width

	Applied in low risk state	Applied in high risk state
<160 ms	0.945 (T=61108, n=10)	0.044 (T=75956, n=10)
160 ms	0.960 (T=392187, n=40)	0.044 (T=352267, n=40)
>160 ms	0.973 (T=235484, n=23)	0.040 (T=156779, n=24)

(D) Burst duration

	Applied in low risk state	Applied in high risk state
<100 ms	0.987 (T=35584, n=3)	0.017 (T=26344, n=3)
100 ms	0.967 (T=562193, n=43)	0.042 (T=438400, n=43)
(100,500] ms	0.929 (T=89671, n=12)	0.053 (T=118706, n=12)
>500 ms	0.993 (T=1331, n=1)	0.005 (T=1552, n=1)

Supplementary Figure S1. Responsive neurostimulation (RNS) settings include (A) stimulation configuration, which specifies how charge flows, and (2) settings across two bursts per each of five therapies, including frequency, amplitude, pulse width, and burst duration.

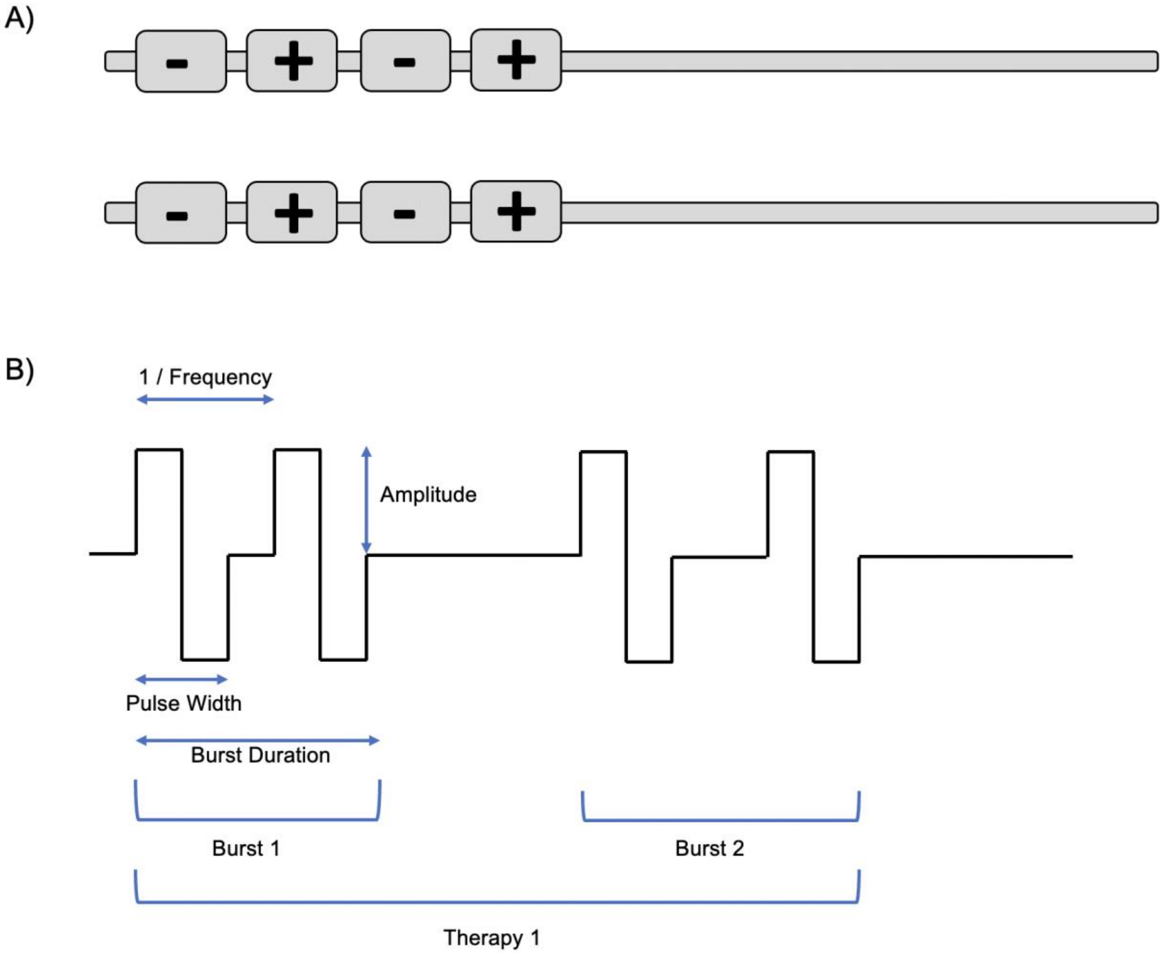

Supplementary Figure S2. Average values of RNS stimulation parameters used in first therapy for (a) monopolar/lead-to-lead stimulation, and (b) bipolar stimulation. Percentage of patient-hours with each stimulation value is shown. Burst duration is shown on log scale.

A)

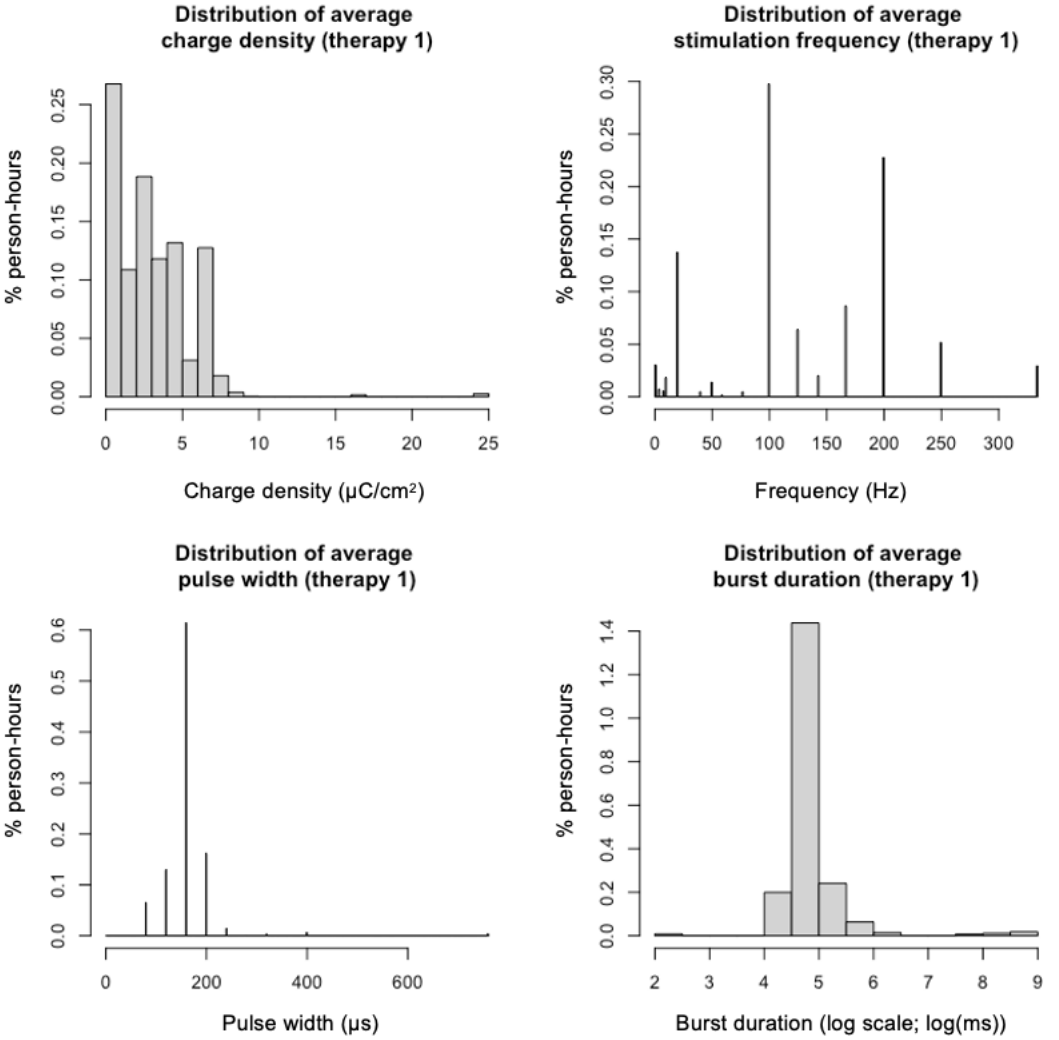

B)

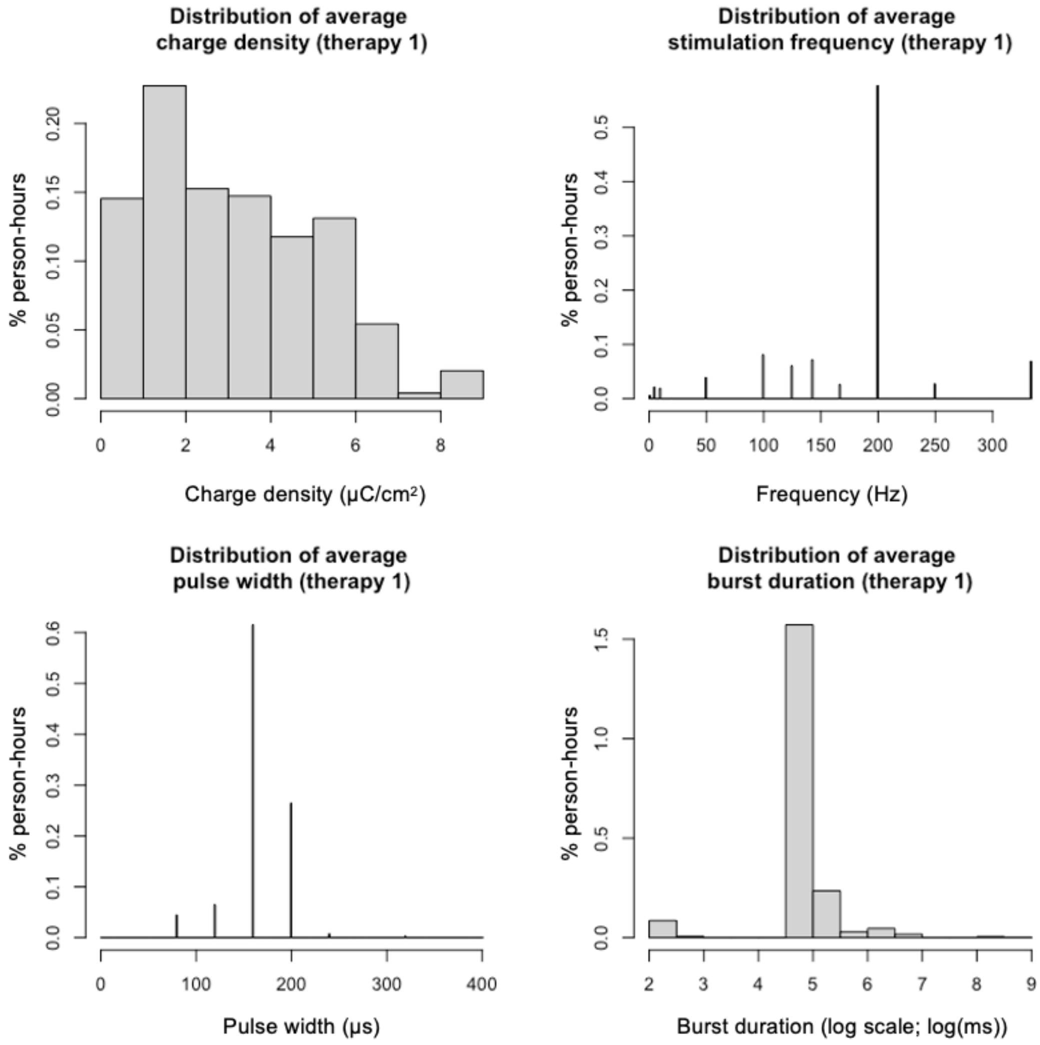
Distribution of average
charge density (therapy 1)
% person-hours
0.00
0.05
0.10
0.15
0.20
0
2
4
6
8
Charge density (μC/cm2)
Distribution of average
stimulation frequency (therapy 1)
% person-hours
0.0
0.1
0.2
0.3
0.4
0.5
0
50
100
150
200
250
300
Frequency (Hz)
Distribution of average
pulse width (therapy 1)
% person-hours
0.0
0.1
0.2
0.3
0.4
0.5
0.6
0
100
200
300
400
Pulse width (μs)
Distribution of average
burst duration (therapy 1)
% person-hours
0.0
0.5
1.0
1.5
2
3
4
5
6
7
8
9
Burst duration (log scale; log(ms))
